# Integrating External Controls by Regression Calibration for Genome-Wide Association Study

**DOI:** 10.3390/genes15010067

**Published:** 2024-01-03

**Authors:** Lirong Zhu, Shijia Yan, Xuewei Cao, Shuanglin Zhang, Qiuying Sha

**Affiliations:** Department of Mathematical Sciences, Michigan Technological University, Houghton, MI 49931, USA; lirongz@mtu.edu (L.Z.); shijiay@mtu.edu (S.Y.); xueweic@mtu.edu (X.C.); shuzhang@mtu.edu (S.Z.)

**Keywords:** genome-wide association test, case-control study, batch effect, data integration

## Abstract

Genome-wide association studies (GWAS) have successfully revealed many disease-associated genetic variants. For a case-control study, the adequate power of an association test can be achieved with a large sample size, although genotyping large samples is expensive. A cost-effective strategy to boost power is to integrate external control samples with publicly available genotyped data. However, the naive integration of external controls may inflate the type I error rates if ignoring the systematic differences (batch effect) between studies, such as the differences in sequencing platforms, genotype-calling procedures, population stratification, and so forth. To account for the batch effect, we propose an approach by integrating External Controls into the Association Test by Regression Calibration (iECAT-RC) in case-control association studies. Extensive simulation studies show that iECAT-RC not only can control type I error rates but also can boost statistical power in all models. We also apply iECAT-RC to the UK Biobank data for M72 Fibroblastic disorders by considering genotype calling as the batch effect. Four SNPs associated with fibroblastic disorders have been detected by iECAT-RC and the other two comparison methods, iECAT-Score and Internal. However, our method has a higher probability of identifying these significant SNPs in the scenario of an unbalanced case-control association study.

## 1. Introduction

Genome-wide association studies (GWASs) play a major role in associating specific genetic variants with continuous or dichotomous phenotypes [1,2,3]. Sometimes, researchers may have limited access to individuals’ genetic information regarding specific phenotypes, and large-scale genetic studies can be expensive and resource-intensive [4]. Thus, with a small sample size in a GWAS, an association test could have low power and may also increase the possibility of false-positive findings, especially for infrequent variants (i.e., minor allele frequency (MAF) < 5%), where MAF refers to the frequency at which the less common allele occurs in a given population [5,6].

The rapid development of sequencing technologies has promoted substantial advancement in GWASs, particularly in obtaining comprehensive genetic information from limited samples [7,8]. This advancement provides an opportunity to enhance the power of single-variant association tests in case-control studies, with several approaches having been proposed. Firstly, the utilization of time-to-event data in case-control studies provides valuable insights into timing and dynamics of events. However, this approach may lead to a loss of information compared to cohort studies due to potential censoring, where some individuals do not experience the event of interest by the end of the study or analysis. Secondly, the integration of sequenced samples from internal and external sources provides a great opportunity for identifying novel genetic associations and increasing the statistical power of single-variant association tests [9]. Specifically, internal sources encompass data generated or collected within the study, which typically include genotype data from genotyping arrays or sequencing platforms, and external sources refer to data obtained from outside the immediate study, such as the utilization of diverse sequencing platforms, variations in genotype-calling procedures, the presence of population stratification, and so forth. Nevertheless, the integration of sequenced samples from internal and external studies is challenging [10]. In a single study, by incorporating sequenced samples from other studies as an external control sample, the power of single-variant tests can be significantly increased without incurring additional sequencing costs. However, the systematic differences (batch effect) arise from various sources, such as different genotyping arrays or sequencing platforms. Integrating sequenced samples from internal and external studies without accounting for these batch effects could inflate type I error rates and increase the possibility of false-positive findings in association studies [11].

Recently, several likelihood-based methods have been proposed to tackle the systematic differences between internal genotyped data and external genotyped data [12]. Liu and Leal proposed the SEQCHIP method to correct bias when integrating genotype data in rare-variant association studies [13]. Derkach et al. proposed another method that substitutes the genotype calls with the expected values given by observed sequence data to account for differential read depths between studies [14]. Chen and Lin proposed regression calibration (RC) methods aimed at addressing the differential sequencing errors between cases and controls [15]. Despite these powerful methods, the calculation of genotype probabilities and the management of sequence read data are challenging in terms of both complexity and cost, particularly in large-scale genetic studies. Therefore, the Proxy External Controls Association Test (ProxECAT) only utilizes allele frequencies of internal cases and external controls to estimate the enrichment of rare variants within a gene [16]. However, the absence of internal controls potentially limits the power of the association test. In contrast, the Integrating External Controls into Association Test (iECAT) uses allele counts from internal cases, internal controls, and external controls to conduct the rare-variant association test [11]. Subsequently, a Bayesian approach is employed to assess the presence of batch effects by comparing the odds ratio estimates between internal controls and combined controls of internal and external studies. External controls that are not subject to batch effects are then integrated with internal samples to increase the sample size. It has been demonstrated that this method can control type I error rates, as well as improve the power of the association test. However, this method cannot adjust for covariates such as age, gender, and so on [11]. Based on the aforementioned method, Li and Lee proposed a novel score-based test that constructs a shrinkage score statistic using internal samples and external control samples, allowing for covariate adjustment for region-based tests [17]. However, the power increase of this method in association testing by integrating external controls is limited for extremely unbalanced case-control studies.

In this study, we present a novel approach that integrates External Controls into Association Tests by Regression Calibration (iECAT-RC) to incorporate external control samples in case-control studies. The objective of this research is to boost the statistical power of the single-variant association test by integrating external controls with the adjustment of batch effects. Our approach adjusts the genotypes of an external control sample to approximate the same distribution as that of the genotypes in the internal control sample through regression calibration. Furthermore, we apply the saddlepoint approximation [18] and efficient resampling [19] methods to control type I error rates with imbalanced case-control and low minor allele count (MAC) scenarios, respectively.

## 2. Materials and Methods

A dichotomous phenotype with case and control states was considered. A case is represented by an individual exhibiting a specific characteristic, which was coded as 1, whereas a control is an individual who does not exhibit this characteristic, which was coded as 0. It was assumed that the internal study had the sample size $n^{I}$ with $n_{0}^{I}$ controls and $n_{1}^{I}$ cases and $n_{0}^{I}+n_{1}^{I}=n^{I}$; the external study had $n_{0}^{E}$ controls. For the $i^{th}$ subject, let $y_{i}=0∕1$ be the dichotomous phenotype. $G_{1},G_{2},\ldots ,G_{n_{0}^{I}},G_{n_{0}^{I}+1},G_{n_{0}^{I}+2},\ldots ,G_{n^{I}}$, and $g_{1},g_{2},\ldots ,g_{n_{0}^{E}}$ are denoted as the genotypes of the internal control sample, the internal case sample, and the external control sample at a genetic variant, respectively, indicating the number of copies of the minor allele carried by the subject at that genetic variant, which can take values of 0, 1, or 2. $X_{i}^{I}$ is the first p principal components of the internal genotypes, and $X_{i}^{E}$ is the first p principal component of the external genotypes for the $i^{th}$ subject. p=10 was used in our simulation studies and real data analysis [20].

Motivated by the novel iECAT-Score method [21], we propose a new method by integrating external controls into association tests to boost the statistical power. Our proposed method involves three steps: (1) adjusting the genotypes of external controls using regression calibration, (2) Conducting a single-variant association test, and (3) calibrating the single-variant test using the saddlepoint approximation (SPA) [18] and efficient resampling (ER) methods [19]—in particular, addressing scenarios of imbalanced case-control and low MAC, respectively. By following these three steps, the iECAT-RC method effectively minimizes the impact of batch effects and improves the power of the single-variant association test.

*Step 1. Adjusting the Genotypes of External Controls by Regression Calibration*

To adjust the genotype of external control samples for the batch effect, we propose using the following procedure:

(1) Without loss of generality, $n_{0}^{E}\geq n_{0}^{I}$ is assumed. A total of $n_{0}^{I}$ individuals with genotypes $g_{k1},\ldots ,g_{kn_{0}^{I}}$ is chosen from external control samples.
(2) A linear regression model $G_{i}=\beta_{0}^{\left(k\right)}+\beta_{1}^{\left(k\right)}g_{ki}+\alpha_{I}^{\left(k\right)}X_{i}^{I}+\alpha_{E}^{\left(k\right)}X_{ki}^{E}$ is assumed for $i=1,\ldots ,n_{0}^{I}$, where ${\hat{\beta }}^{\left(k\right)}={\left({\hat{\beta }}_{0}^{\left(k\right)},{\hat{\beta }}_{1}^{\left(k\right)},{\hat{\alpha }}_{I}^{\left(k\right)},{\hat{\alpha }}_{E}^{\left(k\right)}\right)}^{T}$ is the least-square estimate of $\beta^{\left(k\right)}={\left(\beta_{0}^{\left(k\right)},\beta_{1}^{\left(k\right)},\alpha_{I}^{\left(k\right)},\alpha_{E}^{\left(k\right)}\right)}^{T}$.
(3) (1) and (2) are repeated K times. ${\hat{\beta }}^{\left(1\right)},\ldots ,{\hat{\beta }}^{\left(K\right)}$ are obtained and the average value $\hat{\beta }={\left({\hat{\beta }}_{0},{\hat{\beta }}_{1},{\hat{\alpha }}_{I},{\hat{\alpha }}_{E}\right)}^{T}=\sum_{k=1}^{k}{\hat{\beta }}^{\left(k\right)}∕K$ is calculated. Let $G_{n^{I}+i}={\hat{\beta }}_{0}+{\hat{\beta }}_{1}g_{i}+{\hat{\alpha }}_{I}X_{i}^{I}+{\hat{\alpha }}_{E}X_{i}^{E}$ for $i=1,\ldots ,n_{0}^{I}$. When $G_{n^{I}+i}<a_{0}$, let $G_{n^{I}+i}$ be 0, where $a_{0}$ is determined such that the frequency of 0 in the internal control genotypes is equal to the frequency of 0 in $G_{n^{I}+i}$ for $i=1,\ldots ,n_{0}^{I}$. When $a_{0}\leq G_{n^{I}+i}<a_{1}$, let $G_{n^{I}+i}$ be 1, where $a_{1}$ is determined such that the frequency of 1 in the internal control genotypes is equal to the frequency of 1 in $G_{n^{I}+i}$ for $i=1,\ldots ,n_{0}^{I}$. When $G_{n^{I}+i}>a_{1}$, let $G_{n^{I}+i}$ be 2.

The above procedure is repeated till $G_{n^{I}+i}$ is obtained for $i=1,\ldots ,n_{0}^{E}$. Then, the association test is performed based on the internal case-control data and external control data with genotypes $G_{1},G_{2},\ldots ,G_{n_{0}^{I}},G_{n_{0}^{I}+1},G_{n_{0}^{I}+2},G_{n^{I}},G_{n^{I}+1},\ldots ,G_{n^{I}+n_{0}^{E}}$.

*Step 2. Single-Variant Association Test*

The adjusted genotypes of the internal and external studies are integrated. Let $G={\left(G_{1},G_{2},\ldots ,G_{n}\right)}^{T}$ be the genotype vector at an interested variant for n subjects, where $n=n^{I}+n^{E}$. It is assumed that there is a total of q covariates; then, the phenotype $Y_{i}$ is linked to the covariate $Z_{i}$ and genotype $G_{i}$ using the logistic regression model $\mathrm{logit}\left[P\left(Y_{i}=1|Z_{i},G_{i}\right)\right]=Z_{i}^{T}\alpha +G_{i}\beta$, where the phenotype $Y_{i}$ follows a Bernoulli distribution. Let α be a q×1 coefficient vector for q covariates and include the intercept. Let β be the genotype effect at the variant. Then, the association between the phenotype and the genotype at a variant is evaluated, equivalent to testing $H_{0}:\beta =0$.

Let $\mu =\left\{\mu_{i}\right\}=\left\{P\left(Y_{i}=1|Z_{i}\right)\right\}$ and ${\hat{\mu }}_{i}$ be the maximum-likelihood estimate of $\mu_{i}$ under $H_{0}$. In the score test, the score is given by $S={\tilde{G}}^{T}\left(Y-\hat{\mu }\right)$, Where $Y={\left(Y_{1},\ldots ,Y_{n}\right)}^{T}$, $\tilde{G}=\left\{{\tilde{G}}_{i}\right\}=G-Z{\left(Z^{T}VZ\right)}^{-1}Z^{T}VG$**,** and $V=diag\left\{{\hat{\mu }}_{i}\left(1-{\hat{\mu }}_{i}\right)\right\}$ [2]. Assuming there is no genetic effect under the null hypothesis, E(S)=0 and $Var\left(S\right)=\sum_{i=1}^{n}{\tilde{G}}_{i}^{2}{\hat{\mu }}_{i}\left(1-{\hat{\mu }}_{i}\right)$. Then, the score test statistic $T_{Score}=S^{2}∕Var\left(S\right)$ asymptotically follows the chi-square distribution with 1 degree of freedom, and the *p*-value can be obtained as $p=P\left(\chi_{1}^{2}>S^{2}∕Var\left(S\right)\right)$.

*Step 3. Calibrating Single-Variant Test Using the SPA and ER Methods*

The single-variant score test statistic approximately follows the normal distribution under the null hypothesis. For balanced case-control studies with common variants, variance estimates derived from this asymptotic test behave well. However, when the case-control ratio is not balanced or the MAC is low, leading to extremely low allele frequencies, the underlying distribution of the test statistic may be highly skewed. Thus, the conventional asymptotic score test underperforms in such scenarios and may produce conservative or anticonservative results [22,23].

To account for the scenarios of unbalanced case-control ratio, the SPA method is applied to obtain the *p*-value [18]. When the MAC is low (MAC<10), the ER method is used to obtain the *p*-values [19].

*(1). SPA Method*

SPA is an improvement over normal approximation, which only uses the mean and variance to approximate the underlying distribution. SPA uses the entire cumulant-generating function (CGF). Given the score test statistic $S=\sum_{i=1}^{n}{\hat{G}}_{i}\left(Y_{i}-{\hat{\mu }}_{i}\right)$, the estimation of the CGF of S is $K(t)=\log(E_{H_{0}}(e^{ts}))=\sum_{i=1}^{n}\log(1-{\hat{\mu }}_{i}+{\hat{\mu }}_{i}e^{{\hat{G}}_{i}t})-t\sum_{i=1}^{n}{\hat{G}}_{i}{\hat{\mu }}_{i}$. According to the SPA method, the distribution of S can be estimated by

$$Pr\left(S<s\right)\approx \tilde{F}\left(s\right)=\Phi \left\{\omega +\frac{1}{\omega }\log\left(\frac{\nu }{\omega }\right)\right\},$$

where $\omega =sgn\left(\hat{t}\right)\sqrt{2\left(\hat{t}s-K\left(\hat{t}\right)\right)}$, $\nu =\hat{t}\sqrt{K^{\prime \prime }\left(\hat{t}\right)}$, $K^{\prime }\left(t\right)$, and $K^{\prime \prime }\left(t\right)$ are the estimations of the first- and second-order derivatives of $K;\;\hat{t}$ is the solution to the equation $K^{\prime }\left(\hat{t}\right)=s$; and Φ is the distribution of a standard normal distribution [18]. The *p*-value can be obtained using the R package SPA test.

*(2). ER Method*

The ER method is used for rare-variant association tests with binary traits. Given phenotype Y, genotype G, and covariate Z, the *p*-value of the ER method is defined as

$$\mathrm{Pr}\left(Q\geq \hat{Q}|Y,G,Z\right)=\sum_{d=0}^{m}\mathrm{Pr}\left(Q\geq \hat{Q}|D=d,Y,G,Z\right)\mathrm{Pr}\left(D=d|Y,G,Z\right)$$

where $\hat{Q}$ is the test score statistic from the original phenotype, m is the number of individuals with minor alleles, and D is the number of cases among m individuals carrying a minor allele [19]. The *p*-value can be obtained using the R package SKAT.

## 3. Simulations

In order to evaluate the performance of the proposed iECAT-RC method in terms of the type I error rates and power, we carried out simulation studies under a series of scenarios. We generated the binary phenotypes with cases and controls from a logistic regression model $\mathrm{logit}\left[P\left(Y=1|Z,G\right)\right]=\alpha_{0}+0.5Z_{1}+0.5Z_{2}+\beta G+\varepsilon$, where $Z_{1}$ is a continuous covariate generated from the standard normal distribution, $Z_{2}$ is a binary covariate taking values of 0 and 1 with a probability of 0.5, $\alpha_{0}$ is chosen such that the disease prevalence is 0.05, G is the genotype at a variant generated from a binomial distribution BIN(2,MAF), β is the effect size of the variant, and ε follows a standard normal distribution. MAF was sampled from the empirical Mini-Exome genotype data provided by GAW17, which includes 24,487 variants in 3205 genes, as introduced in Sha et al. [2].

To simulate the batch effect between internal and external control studies, we first defined the differential variant size (DVS) as the proportion of variants with different MAFs between the internal and external control samples. For these variants, we randomly generated the MAFs of the external controls based on two scenarios to mimic the degree of the batch effect: (1) Uniform(0.1q,4q) and (2) 2q, where q is the MAF of the corresponding variants in the internal sample. Subsequently, we considered different numbers of cases and controls in the internal sample and the number of controls in the external controls. We set the following three ratios between the internal cases, internal controls, and external controls $\left(n_{1}^{I}:n_{0}^{I}:n_{0}^{E}\right)$: (1) 5000:5000:10,000, (2) 6667:3333:10,000, and (3) 500:5000:10,000, respectively. Thus, we considered a total of six models. Model 1: the ratio $\left(n_{1}^{I}:n_{0}^{I}:n_{0}^{E}\right)$ is 5000:5000:10,000 and the MAF of the external sample is from 2q; Model 2: the ratio is 6667:3333:10,000 and the MAF of the external sample is from 2q; Model 3: the ratio is 500:5000:10,000 and the MAF of the external sample is from 2q; Model 4: the ratio is 5000:5000:10,000 and the MAF of the external sample is from Uniform(0.1q,4q); Model 5: the ratio is 6667:3333:10,000 and the MAF of the external sample is from Uniform(0.1q,4q); and Model 6: the ratio is 500:5000:10,000 and the MAF of the external sample is from Uniform(0.1q,4q).

We compared our proposed method, iECAT-RC, with three other approaches for the single-variant association test: iECAT-N, which integrates internal and external control samples naïvely; Internal, which uses only the internal sample; and iECAT-Score, as proposed by Li and Lee [21]. If the case-control ratio of the combined sample was unbalanced or the MAC was low (<10 was used in the simulation studies), iECAT-RC, iECAT-N, and Internal used SPA or ER to obtain the corresponding *p*-values, respectively.

To evaluate type I error rates, phenotypes were generated with β=0. For each simulation, we generated $5\times 10^{5}$ data sets and used different significance levels 0.05, 0.01, $10^{-3}$, and $10^{-4}$ for single-variant tests. To save computation time, we generated $5\times 10^{3}$ genotypes and then resampled the disease phenotypes of internal samples 100 times for each set while keeping the other data fixed in the type I error rate evaluation.

To evaluate the power, the effect size β in Model 3 and Model 6 was set as log(2),log(2.4),log(2.8),and log(3.2). The effect size β for other models was set as log(1.6),log(1.8),log(2.0),and log(2.2). We generated $5\times 10^{3}$ data sets for each model to evaluate the empirical power at the significance level of $5\times 10^{-8}$.

## 4. Result

### 4.1. Type I Error Rates

To evaluate the type I error rates, we simulated $5\times 10^{5}$ data sets under the null hypothesis of no association. Table 1 and Table S1 provide a summary of the type I error rates of the four methods—iECAT-RC, iECAT-N, Internal, and iECAT-Score—at different significance levels under DVS=0.03 and 0.5, respectively. From these two tables, we can see that iECAT-RC, Internal, and iECAT-Score controlled type I error rates very well. However, the type I error rates of iECAT-N were significantly inflated when the internal samples and external control samples were naively integrated without adjusting the batch effect. For instance, as shown in Table 1, the empirical type I error rates of iECAT-N exceeded the nominal significance level $\alpha =10^{-4}$ by approximately 1000-fold when the internal and external samples were combined naively. Furthermore, we examine scenarios when the case, control, and external control ratio remained the same but the batch-effect levels differed (Model 1 and Model 4). The performance of the four methods under Model 4 was consistent with those in Model 1. Under both models, the results show well-controlled type I error rates across all methods except iECAT-N. Additionally, we considered scenarios with varying case, control, and external control ratios but the same batch-effect level (Models 1–3). In these cases, iECAT-RC effectively controlled the type I error rates, even under extremely unbalanced case-control samples.

### 4.2. Power

**Figure 1:**
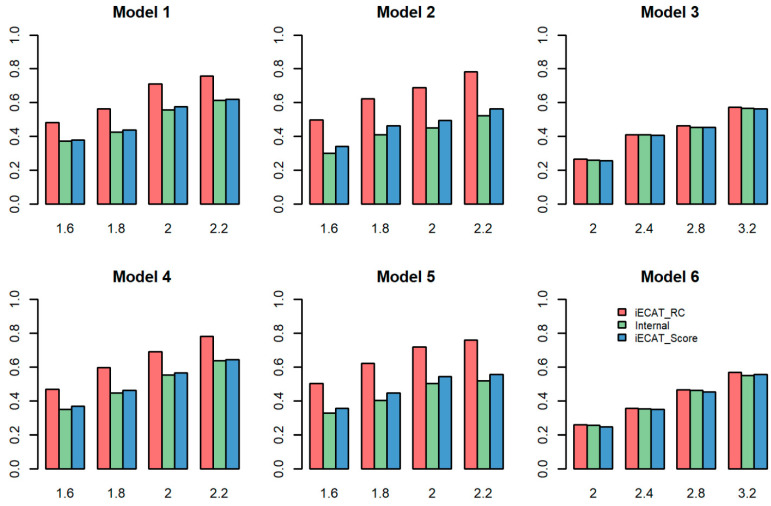
Power comparison of iECAT-RC, Internal, and iECAT-Score at the significance level of $5\times 10^{-8}$ and DVS=0.03. iECAT-N is not considered in power comparison since it is unable to control type I error rates across all scenarios. The horizontal axis represents the odds ratio, and the vertical axis represents power.

To evaluate the performance of our proposed method, we considered different batch-effect levels, different values of DVS, and different values of $n_{1}^{I}:n_{0}^{I}:n_{0}^{E}$. We compared the power of the three methods of iECAT-RC, Internal, and iECAT-Score at an empirical significance level of $5\times 10^{-8}$. iECAT-N was ignored in the power comparison since this method inflates type I error rates. Figure 1 shows the power comparison of these three tests (iECAT-RC, Internal, and iECAT-Score) for different values of $n_{1}^{I}:n_{0}^{I}:n_{0}^{E}$ when the DVS was 0.03. As shown in the figure, in the case of both balanced (Model 1 and Model 4) and slightly unbalanced (Model 2 and Model 5) case-control ratios in the internal samples, iECAT-RC was more powerful than the other two tests; Internal was the least powerful method due to the smaller sample size compared with the other two methods. For the extremely unbalanced internal case-control ratio (Model 3 and Model 6), these three methods had a similar power performance. This is reasonable, because there was slight inflation in the *p*-value for the extremely unbalanced case-control ratio after calibrating the test score via SPA [18].

The power comparison of the three tests for DVS=0.5 is shown in Figure S1. The power patterns of the three methods were very similar between the two different DVS settings for Models 1, 2, 4, and 5. iECAT-RC was more powerful than the other two methods, iECAT was the second powerful method, and Internal was the least powerful method. For Models 3 and 6, similar to the pattern for DVS=0.03, iECAT-RC and Internal had similar power, but iECAT-Score had lower power than iECAT-RC and Internal.

### 4.3. Application to the UK Biobank Data

The UK Biobank dataset, which contains approximately 500,000 individuals with 784,256 variants from across the United Kingdom, provides a prospective cohort for studies aiming to discover more genetic associations and the genetic bases of complex traits with deep genetic and phenotypic data [24,25,26]. In the UK Biobank dataset, genotypes are assayed using two genotype-calling procedures, which are the Applied Biosystems UK BiLEVE Axiom Array (UKBL) and the UK Biobank Axiom Array (UKBB) [27,28]. However, the common practice of calling underlying genotypes and then treating the called values is known to be prone to false-positive findings, especially when genotyping errors are systematically different between cases and controls [29]. Therefore, we applied our proposed method to the real data from the UK Biobank based on two genotype-calling procedures and considered genotype calling as the batch effect. The genotype quality control was performed by PLINK 1.9 https://www.cog-genomics.org/plink/1.9/ (accessed on 2 February 2020) with a missing rate of 5%, a Hardy–Weinberg equilibrium exact test threshold of $10^{-6}$, and a MAF greater than 5% [30]. Then, 288,647 variants were obtained after quality control. We considered the M72 fibroblastic disorders as the phenotype and chose individuals from the UKBL as internal data with 229 cases and the UKBB with controls as the external data. The overlapping variants in these two samples were used in real analysis. The covariate age and sex and the first 10 principal components were adjusted in the model. The descriptive statistics of the subjects from the internal and external studies are shown in Table 2.

**Figure 2:**
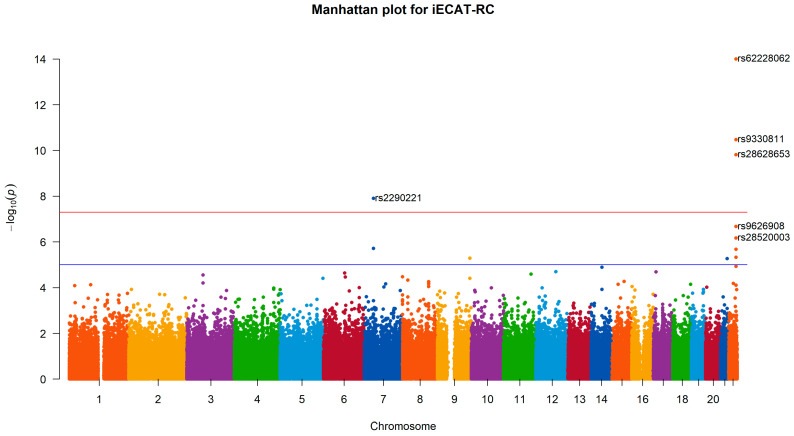
Manhattan plot for M72 fibroblastic disorders based on iECAT-RC. The *p*-values are represented in genomic order by chromosome and position on the chromosome. The value on the *y*-axis represents the −log10 of the *p*-value. This plot is based on 22,701 individuals from the UKBL and 297,068 individuals from the UKBB. The genome-wide significance level is set at $5\times 10^{-8}$. The most significant SNP in the experiment is rs62228062 in the WNT7B gene.

We applied iECAT-RC, Internal, and iECAT-Score to analyze M72 fibroblastic disorders for two genotype-calling procedures in the UK Biobank. Four SNPs were detected to be associated with fibroblastic disorders by all three methods at the significance level of $5\times 10^{-8}$ (Table 3 and Figure 2). iECAT-RC detected these three SNPs with smaller *p*-values. Among the four SNPs, SNP rs62228062 was located in gene WNT7B. A recent transcriptome study identified WNT7B as being amongst the most enriched transcripts in anterior capsule tissue in patients undergoing arthroscopic capsulotomy surgery for frozen shoulder (a tissue disorder), suggesting WNT7B as a potential causal gene at the locus [31]. SNP rs2290221 on chromosome 7 was identified as being associated with fibroblastic disorders and showed the strongest association signal, with a *p*-value of $1.26\times 10^{-8}$, by iECAT-RC. This SNP is in the intronic of genes secreting frizzle-related protein 4 (SFRP4) and ependymal-related protein 1 (zebrafish) (EPDR1). It was detected as being associated with Dupuytren’s disease, which has a large overlap with frozen shoulder-associated loci [31,32].

**Figure 3:**
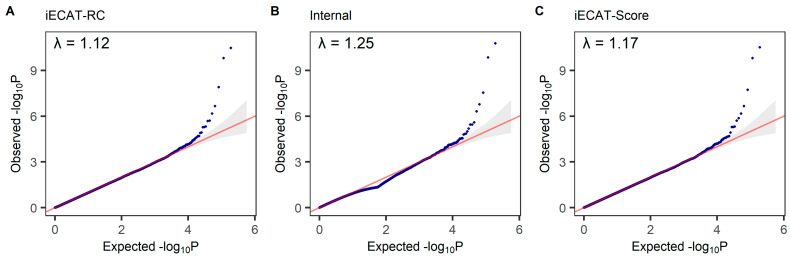
Quantile–quantile (QQ) plot of GWAS results based on iECAT-RC, Internal, and iECAT-Score. The QQ plots show the distribution of the expected *p*-value under the null model versus the observed *p*-value on the −log10 scale. λ indicates the genomic inflation factor.

The Q-Q plot was used to assess the number and magnitude of observed associations between SNPs and the disease under study compared to the association statistics expected under the null hypothesis of no association. The −log10 *p*-values calculated from each method were ranked in order from smallest to largest on the *y*-axis and plotted against the distribution that would be expected under the null hypothesis of no association on the *x*-axis. We tested for association between the disease status of M72 fibroblastic disorders and an SNP, adjusting for age, sex, and the first 10 principal components. The QQ plots from the tests integrating external control samples using the iECAT-RC method, Internal method, and iECAT-Score method are shown in Figure 3. We observed a similarity in patterns among the three QQ plots, all of which closely aligned with the 45 degree line. This alignment indicates that all three methods effectively controlled type I error rates in this analysis.

The case-control ratio of the combined samples had a significant impact on the performance of these three methods (iECAT-RC, Internal, and iECAT-Score), particularly in extremely unbalanced case-control studies, as observed in the simulation studies. Our method demonstrated increased statistical power when the case-control ratio was small. To assess the model’s performance in real data analysis, we randomly selected a subset from the real dataset while maintaining a value of $n_{1}^{I}:n_{0}^{I}:n_{0}^{E}$ is 1:1:2. This allowed us to compare the probabilities of detecting potentially significant SNPs using different methods. Specifically, we conducted 10,000 random samples, with each sample comprising 229 internal cases, 229 internal controls, and 458 external controls. Then, we implemented different methods, and the proportion of detected significant SNPs among the 10,000 samples is presented in Table S2. The proposed method, iECAT-RC, demonstrated a higher probability of detecting significant SNPs. For instance, the relative frequency of detecting SNP rs62228062 was 95.3%, surpassing that of the other two methods.

## 5. Discussion

In case-control studies, it is cost-effective to boost statistical power by increasing the sample size of the case-control study. However, integrating external controls without considering systematic differences (batch effect) between studies, such as the differences in sequencing platforms, genotype-calling procedures, population stratification, and so forth, may lead to inflated type I error rates. In this paper, we propose an approach to integrating external control samples and allow for covariate adjustment. The proposed method, iECAT-RC, effectively addresses potential batch effects by calibrating bias using a regression model.

Simulation studies revealed that iECAT-RC can control for type I error rates very well and boost power in the presence of batch effects. Specifically, we considered different simulation scenarios, including varying the batch-effect level, DVS, and case-control ratios. By comparing iECAT-RC with three referenced methods—Internal, iECAT-Score, and iECAT-N—we demonstrated that all other methods could maintain type I error rates except iECAT-N, which naively combined internal and external samples without adjusting for the batch effects. Additionally, the simulation studies showed that iECAT-RC had a higher power compared with the other methods under different batch-effect mechanisms.

In the real data analysis, we applied iECAT-RC, Internal, and iECAT-Score to genetic data from approximately 500,000 individuals with 784,256 SNPs across the United Kingdom. These individuals were used to identify the association between SNPs and M72 fibroblastic disorders while considering the genotype calling as the batch effect. Although all three methods—iECAT-RC, Internal, and iECAT-Score—identified four SNPs that are significantly associated with the disease, our proposed method had a higher probability of detecting these disease-associated SNPs compared to the other two methods when the case-control ratio was 1:3.

In conclusion, the proposed iECAT-RC method can integrate external control samples and, at the same time, control type I error rate and boost statistical power. Through the linear regression calibration, we effectively reduced the batch effects arising from different platforms. Additionally, we employed the SPA [18] and ER [19] methods to accurately calibrate *p*-values in scenarios of unbalanced case-control ratios and low MAFs. Our method provides a robust and effective improvement in score tests, ultimately contributing to a better understanding of the genetic architecture of complex diseases. However, iECAT-RC has limited power improvement when internal samples have an extremely unbalanced case-control ratio. Furthermore, it is necessary for external samples to originate from the same ancestry to eliminate population stratification.

iECAT-RC is suitable for case-control studies focusing on any dichotomous phenotypes, particularly those influenced by rare variants. Given that rare variants occur at low frequencies within populations, they may not be identified through conventional GWASs. iECAT-RC addresses this limitation by integrating external sequenced data, thereby enhancing the sample size and enabling the detection of associated genetic variants.

## Figures and Tables

**Table 1:** Empirical type I error rates of iECAT-RC compared with the other three methods—iECAT-N, Internal, and iECAT-Score—when DVS is 0.03 at different significance levels of 0.05, 0.01, $10^{-3}$, and $10^{-4}$.

Model	Significance Level	iECAT-RC	iECAT-N	Internal	iECAT-Score
Model 1	0.05	0.0382	**0.3956**	0.0512	0.0482
	0.01	0.0057	**0.3352**	0.0102	0.0096
	0.001	3.00 × 10^−4^	**0.2771**	0.001	0.001
	1 × 10^−4^	1.00 × 10^−4^	**0.2429**	1.00 × 10^−4^	0
Model 2	0.05	0.0397	**0.4163**	0.0348	0.0394
	0.01	0.0078	**0.3685**	0.0087	0.0089
	0.001	9.00 × 10^−4^	**0.3263**	4.00 × 10^−4^	0.0013
	1 × 10^−4^	1.00 × 10^−4^	**0.2919**	0	2.00 × 10^−4^
Model 3	0.05	0.0457	**0.113**	0.0136	0.0357
	0.01	0.0111	**0.0628**	0.004	0.0081
	0.001	6.00 × 10^−4^	**0.0345**	5.00 × 10^−4^	3.00 × 10^−4^
	1 × 10^−4^	0	**0.0223**	0	0
Model 4	0.05	0.0372	**0.4269**	0.0511	0.0475
	0.01	0.0065	**0.3513**	0.0105	0.0101
	0.001	4.00 × 10^−4^	**0.2804**	9.00 × 10^−4^	0.001
	1 × 10^−4^	0	**0.2359**	3.00 × 10^−4^	1.00 × 10^−4^
Model 5	0.05	0.0494	**0.457**	0.0335	0.0446
	0.01	0.0107	**0.3876**	0.0079	0.0096
	0.001	0.0017	**0.3244**	9.00 × 10^−4^	0.001
	1 × 10^−4^	4.00 × 10^−4^	**0.2806**	0	1.00 × 10^−4^
Model 6	0.05	0.0467	**0.1013**	0.0133	0.0342
	0.01	0.011	**0.0569**	0.0042	0.007
	0.001	0.0012	**0.0291**	9.00 × 10^−4^	7.00 × 10^−4^
	1 × 10^−4^	1.00 × 10^−4^	**0.0169**	0	0

Note: The bold-faced values indicate the type I error rates beyond the upbound of the corresponding 95% confidence interval.

**Table 2:** Descriptive statistics of subjects from the UK Biobank for real analysis.

Study	Samples Size		
	Cases	Controls	Totals
UKBL (internal)	229	22,472	22,701
UKBB (external)		297,068	297,068
Total	229	318,540	319,769

**Table 3:** Significant SNPs identified by iECAT-RC, iECAT-Score, and Internal at a significance level of $5\times 10^{-8}$.

Chromosome	SNP	Base Position	Genes	iECAT-RC	iECAT-Score	Internal
7	rs2290221	37987632	SFRP4, EPDR1	1.26 × 10^−8^	2.91 × 10^−8^	1.86 × 10^−8^
22	rs9330811	46362396	WNT7B	1.65 × 10^−11^	3.37 × 10^−11^	3.00 × 10^−11^
22	rs62228062	46381234	WNT7B	6.04 × 10^−18^	8.82 × 10^−18^	6.04 × 10^−18^
22	rs28628653	46396925	LOC730668	1.54 × 10^−10^	1.40 × 10^−10^	1.54 × 10^−10^

## Data Availability

Data are contained within the article and supplementary materials.

## Supplementary Materials

The following supporting information can be downloaded at: https://www.mdpi.com/article/10.3390/genes15010067/s1, Table S1: Empirical type I error rates of iECAT-RC, compared with other three methods iECAT-N, Internal, and iECAT-Score at different significance levels, 0.05, 0.01, 10^−3^, and 10^−4^ with DVS = 0.5; Table S2: The relative frequency of significant SNPs identified by each method using 10,000 repeated samples; Figure S1. The power comparison of iECAT-RC, Internal, and iECAT-Score when DVS = 0.5 at the significance level of 5 × 10^−8^. The horizontal axis represents the odds ratio, and the vertical axis represents power.
