# Supplementary material for: Integrating External Controls by Regression Calibration for Genome-Wide Association Study
Source: Genes (Basel). 2024 Jan 3;15(1):67. doi: 10.3390/genes15010067 (PMC10815702; doi:10.3390/genes15010067)
Supplement: Supplementary file 1 [file genes-15-00067-s001.zip › genes-2797861-supplementary.pdf]

Supplementary Materials

Integrating external controls by regression calibration for genome-wide association study

Lirong Zhu[1], Shijia Yan[1], Xuewei Cao[1], Shuanglin Zhang[1], Qiuying Sha[1,*]

[1]Department of Mathematical Sciences, Michigan Technological University, Houghton, Michigan, USA

[*]Corresponding author: Qiuying Sha, Department of Mathematical Sciences, Michigan Technological University, Houghton, Michigan 49931, USA. E-mail: qsha@mtu.edu

Table S1. Empirical type I error rates of iECAT-RC, compared with other three methods iECAT-N, Internal, and iECAT-Score at different significance levels, $0.05, 0.01, 10^{-3}$ and 10^{-4} with $DVS = 0.5$.

Model	Significance level	iECAT-RC	iECAT-N	Internal	iECAT-Score
Model 1	0.05	0.033	**0.3895**	0.05	0.0457
	0.01	0.0057	**0.3285**	0.0102	0.0077
	0.001	3×10^{-4}	**0.2762**	0.0012	9×10^{-4}
	1×10^{-4}	0	**0.2386**	2×10^{-4}	0
Model 2	0.05	0.0529	**0.4083**	0.0343	0.0449
	0.01	0.0109	**0.357**	0.0089	0.0111
	0.001	7×10^{-4}	**0.3217**	9×10^{-4}	0.0016
	1×10^{-4}	1×10^{-4}	**0.2912**	1×10^{-4}	0
Model 3	0.05	0.0464	**0.1334**	0.0139	0.0377
	0.01	0.0103	**0.0829**	0.0037	0.0066
	0.001	9×10^{-4}	**0.0513**	5×10^{-4}	8×10^{-4}
	1×10^{-4}	0	**0.0364**	0	0
Model 4	0.05	0.0323	**0.4163**	0.042	0.0407
	0.01	0.0052	**0.3292**	0.0089	0.0082
	0.001	6×10^{-4}	**0.2608**	0.0013	4×10^{-4}
	1×10^{-4}	2×10^{-4}	**0.2231**	3×10^{-4}	0
Model 5	0.05	0.0501	**0.475**	0.0327	0.0492
	0.01	0.0117	**0.4002**	0.0097	0.0087
	0.001	0.0016	**0.3289**	0.0011	0.0016
	1×10^{-4}	0	**0.2775**	1×10^{-4}	4×10^{-4}
Model 6	0.05	0.0453	**0.1017**	0.0139	0.0316
	0.01	0.0102	**0.0546**	0.0044	0.0072
	0.001	0.0012	**0.028**	8×10^{-4}	7×10^{-4}
	1×10^{-4}	0	**0.0158**	1×10^{-4}	0

Notes: Type I error rates are evaluated based on 5×10^5 simulations. The bold-faced values indicate the type I error rates beyond the upbound of the corresponding 95% confidence interval.

Table S2. The relative frequency of significant SNPs identified by each method using 10,000 repeated samples.

Chr	SNP	Base Position	iECAT-RC	iECAT-Score	Internal
22	rs62228062	46381234	95.3%	79.5%	37.4%
22	rs9330811	46362396	54.3%	38.8%	3.9%
22	rs28628653	46396925	34.7%	15.2%	1.1%
7	rs2290221	37987632	11.2%	1.8%	0.0%
22	rs9626908	46428306	8.3%	0.7%	0.2%
9	rs12685782	135320210	6.3%	0.2%	0.1%
22	rs28520003	46411969	5.0%	0.2%	0.1%

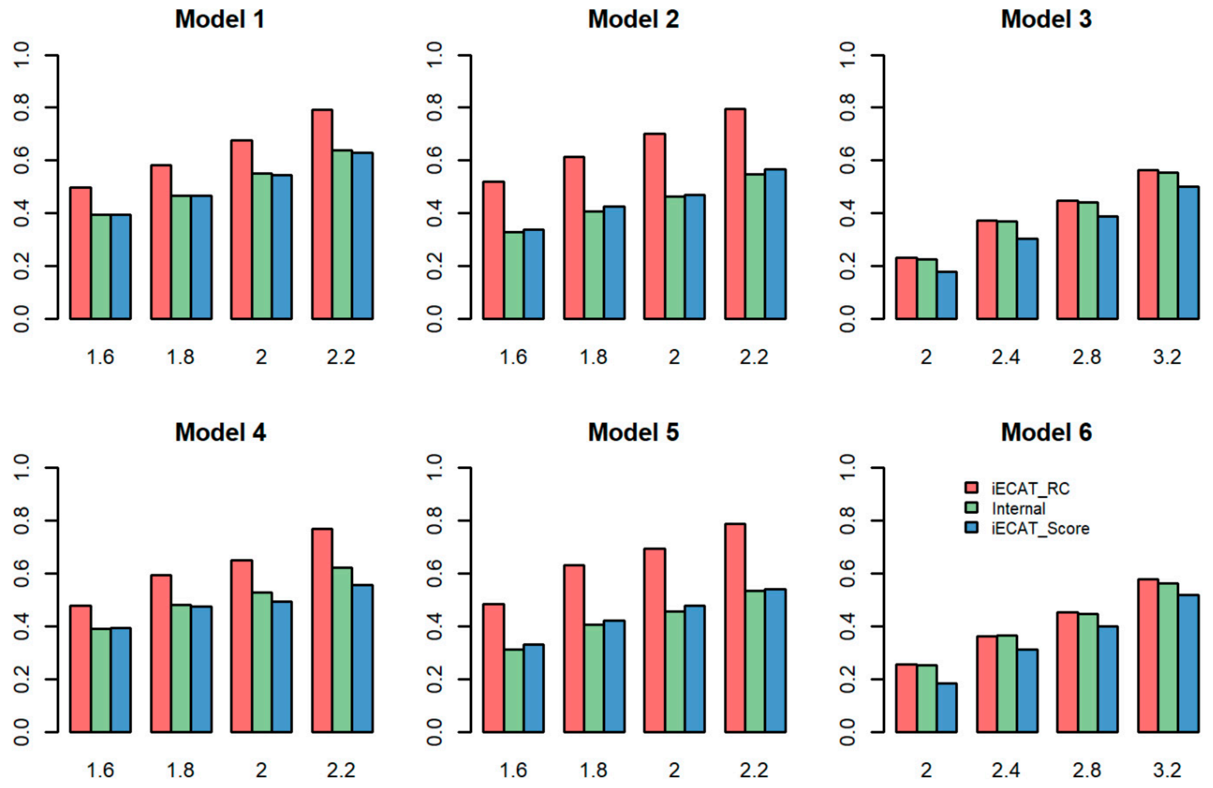

Figure S1. The power comparison of iECAT-RC, Internal, and iECAT-Score when $DVS = 0.5$ at the significance level of 5×10^{-8}The horizontal axis represents the odds ratio, and the vertical axis represents power.
